# Predictive model of acute kidney injury in critically ill patients with acute pancreatitis: a machine learning approach using the MIMIC-IV database

**DOI:** 10.1080/0886022X.2024.2303395

**Published:** 2024-01-24

**Authors:** Shengwei Lin, Wenbin Lu, Ting Wang, Ying Wang, Xueqian Leng, Lidan Chi, Peipei Jin, Jinjun Bian

**Affiliations:** Faculty of Anesthesiology, Changhai Hospital, Naval Medical University, Shanghai, China

**Keywords:** Acute kidney injury, acute pancreatitis, machine learning, prediction model, MIMIC- IV database

## Abstract

**Background:**

Acute kidney injury (AKI) is a common and serious complication in severe acute pancreatitis (AP), associated with high mortality rate. Early detection of AKI is crucial for prompt intervention and better outcomes. This study aims to develop and validate predictive models using machine learning (ML) to identify the onset of AKI in patients with AP.

**Methods:**

Patients with AP were extracted from the MIMIC-IV database. We performed feature selection using the random forest method. Model construction involved an ensemble of ML, including random forest (RF), support vector machine (SVM), k-nearest neighbors (KNN), naive Bayes (NB), neural network (NNET), generalized linear model (GLM), and gradient boosting machine (GBM). The best-performing model was fine-tuned and evaluated through split-set validation.

**Results:**

We analyzed 1,235 critically ill patients with AP, of which 667 cases (54%) experienced AKI during hospitalization. We used 49 variables to construct models, including GBM, GLM, KNN, NB, NNET, RF, and SVM. The AUC for these models was 0.814 (95% CI, 0.763 to 0.865), 0.812 (95% CI, 0.769 to 0.854), 0.671 (95% CI, 0.622 to 0.719), 0.812 (95% CI, 0.780 to 0.864), 0.688 (95% CI, 0.624 to 0.752), 0.809 (95% CI, 0.766 to 0.851), and 0.810 (95% CI, 0.763 to 0.856) respectively. In the test set, the GBM’s performance was consistent, with an area of 0.867 (95% CI, 0.831 to 0.903).

**Conclusions:**

The GBM model’s precision is crucial, aiding clinicians in identifying high-risk patients and enabling timely interventions to reduce mortality rates in critical care.

## Introduction

Worldwide hospital admission rates are significantly influenced by acute pancreatitis (AP), a gastrointestinal condition with a global prevalence affecting various age groups and genders [1, 2]. In the context of AP, acute kidney injury (AKI) frequently emerges as a complication, especially in severe cases, often occurring at an advanced stage and following the deterioration of other vital organs [3]. Despite comprehensive practice guidelines for AP management, morbidity and mortality rates persist stubbornly [4]. Importantly, increased mortality in severe AP cases is closely tied to organ failure and subsequent secondary infections—key factors shaping AP outcomes [5]. Therefore, it is crucial to clinically assess symptoms and indicators of organ failure (including respiratory, cardiovascular, and renal systems) in AP patients to accurately categorize the condition.

Therefore, clinicians face a critical need to early anticipate the likelihood of acute renal injury in patients with acute pancreatitis, a foresight that can significantly guide and improve clinical interventions. Additionally, in the realm of AKI detection, the imperative is for the diagnostic method to possess attributes such as minimal invasiveness, widespread accessibility, cost-effectiveness, procedural simplicity, and replicability [6]. Several studies have explored determinants and established prognostic frameworks to predict AKI onset in individuals with acute pancreatitis. However, these investigations have been hindered by limited sample sizes and a lack of precision necessary for robust prognostic modeling [7]. The challenge of promptly and accurately diagnosing AKI in acute pancreatitis patients persists within clinical practice.

In recent times, there has been a surge in the practical use of powerful computational methodologies, especially within the field of machine learning, for disease prediction efforts. Machine learning (ML), an emerging domain, has increasingly become integral to medical research. It’s important to note that the effectiveness of ML analysis relies on the iterative use of diverse algorithms of varying depth, enabling the assimilation of candidate variables. This approach contributes to achieving prediction efficiency characterized by elevated precision [8].

In line with this cognitive perspective, our focus was on developing a prognostic framework outlining the risk of AKI in acute pancreatitis patients. We utilized the extensive information from the critical care database in this investigation. The envisioned outcome of this predictive model is an instrument capable of prompting quick interventions, thus creating a conducive environment for managing high-risk AKI cases. The prescient identification achieved through the model’s effectiveness plays a pivotal role in the domain of intensive care.

## Methods

### Data source

This study utilized the Medical Information Mart for Intensive Care IV database version 2.2 (MIMIC-IV v2.2) as its primary dataset. MIMIC-IV, a publicly accessible repository of critical care data from a single medical center, has received approval from the Institutional Review Boards of Beth Israel Deaconess Medical Center (BIDMC, Boston, MA, USA) and the Massachusetts Institute of Technology (MIT, Cambridge, MA, USA). The database includes comprehensive records for a cohort of 73,181 patients admitted to various Intensive Care Units at BIDMC in Boston, Massachusetts, covering the period from 2008 to 2019 [9]. The dataset comprises well-documented events, including demographic indicators, vital sign readings, laboratory results, fluid balance assessments, and patient survival status. Additionally, the database includes International Classification of Diseases and Revision (ICD-9 and ICD-10) codes, offering a standardized framework for systematic classification. Notably, the repository includes hourly physiologic data collected from bedside monitors and rigorously validated by skilled ICU nursing personnel.

The content of this database, made available through contributions from clinicians, data scientists, and information technology experts, prioritizes an anonymity-centered approach to protect patients’ health-related information. This orientation has resulted in the exemption of the database from the realm of human subjects research, eliminating the need for individual patient consent due to the anonymized nature of the health data. It is crucial to highlight that potential users undergo a rigorous assessment procedure, including the successful completion of a qualifying examination and obtaining approval from the MIMIC-IV database administration. As an example, Wenbin Lu, an author of this study, completed a mandatory training course, leading to authorization for data extraction from the database for research purposes (certification number: 50992435)

### Patients and data variables

Data extraction was carried out using Structured Query Language (SQL) programming within the PostgreSQL framework (version 14.0). The SQL script codes needed for extracting patient information were obtained from the GitHub repository located at (https://github.com/MIT-LCP/mimic-code/tree/main/mimic-iv) [10], ensuring methodological transparency and replicability.

Patients diagnosed with acute pancreatitis were identified from database using the International Classification of Diseases, ninth revision (ICD-9, code 577.0), and tenth revision (ICD10, code K85%). Prudent exclusion criteria included patients under 18 years of age, those with an ICU stay of fewer than 24 h, and individuals with a documented history of renal disease. For patients with multiple ICU admissions, data retrieval was exclusively performed for the initial admission, ensuring methodological consistency.

After meticulously identifying eligible patient cohorts, a comprehensive set of fundamental parameters characterizing patients with AP was systematically gathered. These parameters included a range of demographic characteristics, relevant medical histories, vital sign recordings, laboratory indices, and instances of interventions. The interventions under consideration included the use of invasive mechanical ventilation, the implementation of renal replacement therapy, and the administration of vasoactive agents. It is important to note that both vital sign measurements and laboratory indicators were based on the initial values recorded during the first 24 h following the patient’s admission to the ICU.

The chosen outcome measure focused on the incidence of AKI within seven days following the patient’s admission to the ICU. To diagnose AKI, we adhered to the guidelines outlined in the 2012 version of the Kidney Disease: Improving Global Outcomes (KDIGO) guidelines. The diagnostic criteria were as follows: an increase in serum creatinine (SCr) levels by ≥26.5 μmol/L (0.3 mg/dl) within a 48-h period; an increase in SCr values by ≥50% compared to the baseline value (resulting in a 1.5-fold increase); or a urinary output less than 0.5 mL/kg/h for more than six hours. Importantly, the baseline SCr, a crucial parameter in this diagnostic framework, is defined as the lowest observed SCr value within the immediate preceding week [11].

## Statistical analysis

### Data cleaning and feature selection

The database exhibited a noteworthy prevalence of missing data. In this study, variables with missing values exceeding a 20% threshold were intentionally excluded. The variables and their corresponding proportions of missing values are thoroughly outlined in Appendix 1. To address these data gaps, multiple imputations were employed, as detailed in reference [12]. Skewed distribution patterns led to the use of median and quartile representations, facilitating a robust Mann–Whitney U test for inter-variable comparisons. Categorical variables were succinctly presented through count (%) depictions and underwent comparative analysis using the χ^2^ test. Feature selection was pragmatically carried out through recursive feature elimination, an inherent aspect of the random forest, supported by the ‘rfe’ function within the ‘caret’ package. This process resulted in the curation of a thoughtfully selected subset of features ready for integration into the subsequent machine-learning models.

### Model selection

**Figure 1.**
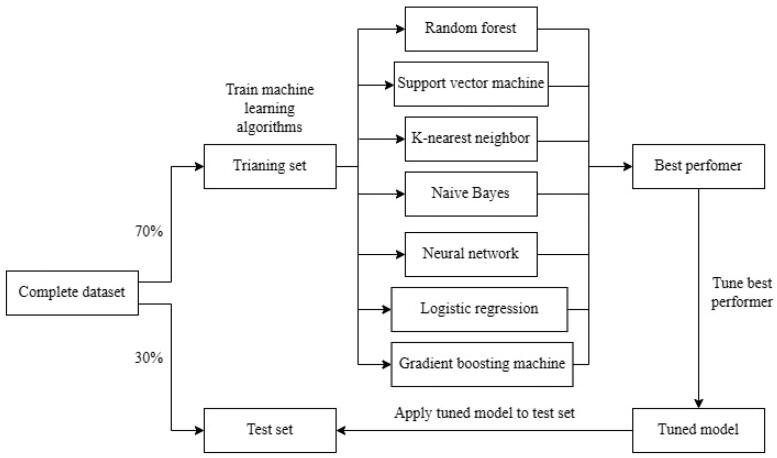
Diagram of methods. The complete data set was split into training and test sets. The machine-learning methods were trained on the training set and the best performer selected for additional parameter tuning before being applied to the test set for validation.

To address the variability in internal validation robustness across different machine-learning methods, a prudent approach involved dividing the data into training and test sets, using a randomized allocation of 70/30 for validation purposes. Specifically, 70% of the dataset was used for the training phase, while the remaining 30% served as a separate test set (Figure 1). In a non-preordained sequence, machine-learning algorithms underwent training within the training set, incorporating a 10-fold cross-validation protocol repeated thrice to mitigate initial tendencies toward overfitting. Additionally, the evaluation of each model included the computation of sensitivity and specificity at the ‘best’ thresholds. It is important to clarify that the term ‘best’ threshold refers to the point at which both sensitivity and specificity simultaneously reach their peak, although not always aligning with the optimal threshold for clinical application.

The array of trained machine-learning algorithms included generalized linear model, support vector machines, naive Bayes, k-nearest neighbor, random forest, neural networks, and stochastic gradient boosting machine. All features surviving recursive feature elimination were uniformly employed across the training algorithms. Certain machine-learning algorithms, such as the random forest, have inherent built-in feature selection capabilities. The initial training and subsequent 10-fold cross-validation were orchestrated using the ‘caret’ package in R, with the receiver operating characteristic as the designated performance metric. Parameter calibration, specific to machine-learning approach, underwent a basic tuning process following defaults set by the package [13–16]. Additionally, receiver operating characteristic curves were generated using the ‘pROC’ package. A comprehensive representation of the model’s confidence intervals was achieved by computing bootstrap-based 95% confidence intervals, obtained through 2,000 stratified bootstrap replicates using the ‘ci’ function within the ‘pROC’ package.

### Model tuning and testing

Following meticulous model selection, characterized by the initial course-level tuning of the models, the subsequent phase involved the careful refinement of the most proficient algorithm. This refinement was based on its achievement of the highest area under the receiver operating characteristic curve, a crucial criterion for identifying the optimal model. The fine-tuning process, indicating the intentional optimization of algorithmic performance through judicious modification of relevant parameters, occurred in alignment with the specific requirements of each machine-learning method. In this pursuit, tuning involved the calibration of method-specific parameters in a conscientious effort to achieve optimal performance benchmarks.

The optimization efforts for these method-specific parameters involved a thorough manual grid search. This rigorous procedure entailed introducing broad yet practical ranges of conceivable values for each parameter, followed by a meticulous comparative analysis of the performance exhibited by the resulting models. For models allowing the calculation of variable importance, this attribute was determined for the final model. It’s important to note that the feasibility of computing variable importance varies across machine-learning algorithms. This quantification, based on the classifier’s construction and its subsequent impact on the performance metric, establishes the significance of individual features in facilitating the classification process.

Variable importance serves as a crucial metric for evaluating the influence of individual variables on the algorithm’s performance. This assessment is notably discerned through the examination of the repercussions resulting from the permutation or omission of a variable with heightened importance from the model, leading to a corresponding decrement in performance. The magnitude of importance established conveys the indispensability of a given variable to the model’s efficacy, underscoring its contribution to the performance outcomes. However, it’s important to highlight that the direct derivation of effect size in relation to the primary outcome based on variable importance remains elusive [17]. The quantification of variable importance was operationalized *via* the ‘varImp’ function, intrinsic to the ‘caret’ package. Subsequently, the ultimate model underwent validation through simulation on the test set, elucidating both the algorithm’s capacity for generalization and the avoidance of overfitting [18]. A visual representation of this process is explained in Figure 1. To assess the model’s discriminative prowess, the area under the receiver operating characteristic curve (AUC) was employed as the yardstick for evaluation. Significance levels were established at *p* < 0.05. The entire analytical process was conducted within the R software framework (version 4.2.2).

## Results

**Figure 2.**
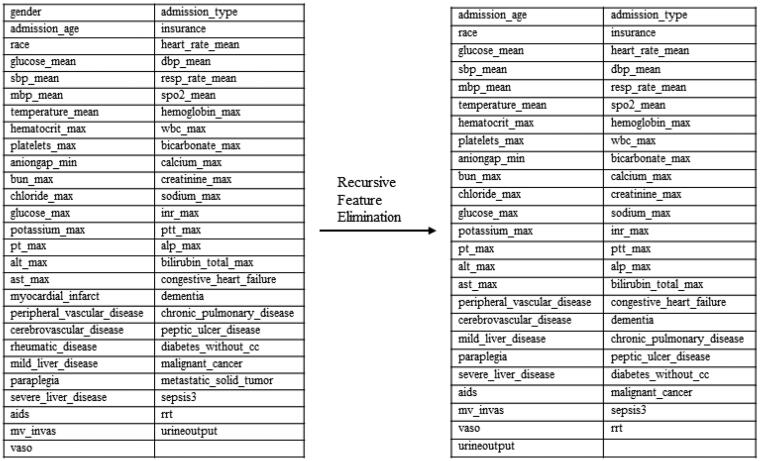
Reduction of dimensionality by recursive feature elimination on the training data set. The number of features used for training was reduced from the list of features on the left to the list of features on the right.

Following the exclusion of patients below the age of 18, the resulting cohort comprised 1,235 individuals diagnosed with acute pancreatitis (AP), of whom 667 (54%) developed acute kidney injury (AKI) within the subsequent 7 days. The training set, consisting of 865 cases, was balanced by the test set, which included 370 cases. In the training set, 467 (53.9%) cases experienced AKI, while the test set recorded 200 (54.0%) instances of AKI. The comprehensive data characteristics of the entire dataset are meticulously outlined in Table 1. The final selection of features following recursive feature elimination is visually depicted in Figure 2.

**Table 1.** Baseline characteristics.

Variable	Overall, *N* = 1,235	No, *N* = 568	Yes, *N* = 667	p-value
gender				0.3
F	547 (44%)	243 (43%)	304 (46%)	
M	688 (56%)	325 (57%)	363 (54%)	
admission_type				0.3
EMERGENCY	677 (55%)	320 (56%)	357 (54%)	
OTHER	558 (45%)	248 (44%)	310 (46%)	
admission_age	59 (46, 72)	56 (45, 68)	61 (49, 74)	<0.001
race				0.3
WHITE	822 (67%)	369 (65%)	453 (68%)	
OTHER	413 (33%)	199 (35%)	214 (32%)	
insurance				0.6
MEDICARE	418 (34%)	188 (33%)	230 (34%)	
OTHER	817 (66%)	380 (67%)	437 (66%)	
glucose_mean	129 (106, 166)	125 (104, 163)	132 (110, 169)	0.003
heart_rate_mean	92 (80, 106)	90 (77, 103)	93 (81, 107)	<0.001
sbp_mean	119 (108, 133)	124 (112, 136)	115 (106, 129)	<0.001
dbp_mean	67 (59, 76)	70 (62, 79)	64 (57, 72)	<0.001
mbp_mean	80 (73, 90)	84 (75, 92)	77 (71, 87)	<0.001
resp_rate_mean	19.6 (16.9, 22.6)	18.9 (16.3, 21.6)	20.2 (17.6, 23.8)	<0.001
temperature_mean	36.90 (36.64, 37.25)	36.89 (36.66, 37.22)	36.90 (36.63, 37.29)	0.8
spo2_mean	96.48 (95.12, 98.00)	96.52 (95.26, 98.08)	96.44 (94.95, 97.97)	0.2
hematocrit_max	35 (31, 40)	34 (31, 39)	35 (31, 40)	0.10
hemoglobin_max	11.60 (10.00, 13.10)	11.70 (10.10, 13.00)	11.60 (10.00, 13.30)	0.7
platelets_max	209 (149, 296)	209 (150, 292)	209 (148, 303)	0.7
wbc_max	13 (9, 18)	11 (8, 15)	15 (10, 20)	<0.001
aniongap_min	13.0 (11.0, 15.0)	13.0 (11.0, 14.0)	13.0 (11.0, 15.0)	0.002
bicarbonate_max	24.0 (21.0, 27.0)	24.0 (22.0, 27.0)	23.0 (20.0, 26.0)	<0.001
bun_max	18 (12, 29)	15 (10, 23)	22 (15, 35)	<0.001
calcium_max	8.36 (7.80, 8.80)	8.40 (7.90, 8.90)	8.30 (7.80, 8.80)	0.010
chloride_max	106 (102, 110)	105 (102, 109)	106 (102, 110)	0.057
creatinine_max	1.00 (0.70, 1.40)	0.90 (0.70, 1.10)	1.10 (0.80, 1.90)	<0.001
glucose_max	146 (115, 204)	137 (111, 187)	154 (119, 212)	<0.001
sodium_max	139.0 (137.0, 142.0)	139.0 (137.0, 142.0)	139.0 (137.0, 143.0)	0.9
potassium_max	4.30 (3.90, 4.80)	4.20 (3.90, 4.50)	4.30 (4.00, 4.90)	<0.001
inr_max	1.40 (1.20, 1.65)	1.30 (1.10, 1.60)	1.40 (1.20, 1.70)	<0.001
pt_max	15 (13, 18)	14 (13, 18)	16 (14, 19)	<0.001
ptt_max	33 (28, 40)	32 (28, 40)	35 (29, 43)	<0.001
alt_max	66 (28, 226)	62 (28, 226)	68 (28, 226)	0.5
alp_max	117 (75, 151)	118 (74, 151)	117 (75, 152)	>0.9
ast_max	93 (40, 389)	81 (37, 370)	107 (44, 389)	0.009
bilirubin_total_max	1.50 (0.60, 2.95)	1.20 (0.60, 2.95)	1.80 (0.70, 3.10)	<0.001
myocardial_infarct	113 (9.1%)	43 (7.6%)	70 (10%)	0.076
congestive_heart_failure	190 (15%)	59 (10%)	131 (20%)	<0.001
peripheral_vascular_disease	73 (5.9%)	26 (4.6%)	47 (7.0%)	0.067
dementia	34 (2.8%)	15 (2.6%)	19 (2.8%)	0.8
cerebrovascular_disease	81 (6.6%)	30 (5.3%)	51 (7.6%)	0.094
chronic_pulmonary_disease	266 (22%)	117 (21%)	149 (22%)	0.5
rheumatic_disease	39 (3.2%)	19 (3.3%)	20 (3.0%)	0.7
peptic_ulcer_disease	65 (5.3%)	42 (7.4%)	23 (3.4%)	0.002
mild_liver_disease	328 (27%)	137 (24%)	191 (29%)	0.073
diabetes_without_cc	321 (26%)	150 (26%)	171 (26%)	0.8
paraplegia	26 (2.1%)	12 (2.1%)	14 (2.1%)	>0.9
malignant_cancer	141 (11%)	64 (11%)	77 (12%)	0.9
severe_liver_disease	128 (10%)	32 (5.6%)	96 (14%)	<0.001
metastatic_solid_tumor	63 (5.1%)	27 (4.8%)	36 (5.4%)	0.6
aids	13 (1.1%)	7 (1.2%)	6 (0.9%)	0.6
sepsis3	676 (55%)	205 (36%)	471 (71%)	<0.001
mv_invas	441 (36%)	94 (17%)	347 (52%)	<0.001
rrt	90 (7.3%)	2 (0.4%)	88 (13%)	<0.001
vaso	361 (29%)	60 (11%)	301 (45%)	<0.001
urineoutput (ml)	1,590 (952, 2,455)	2,075 (1,405, 3,125)	1,232 (760, 1,890)	<0.001

mv_invas: invasive mechanical ventilation; rrt: renal replacement therapy; vaso: vasoactive drugs.

After the completion of training, the area under the receiver operating characteristic curve was computed for each machine-learning method: generalized linear model yielded an AUC of 0.812 (95% CI, 0.769 to 0.854); support vector machines, 0.810 (95% CI, 0.763 to 0.856); naive Bayes, 0.812 (95% CI, 0.780 to 0.864); k-nearest neighbor, 0.671 (95% CI, 0.622 to 0.719); random forest, 0.809 (95% CI, 0.766 to 0.851); neural networks, 0.688 (95% CI, 0.624 to 0.752); and gradient boosting machines, 0.814 (95% CI, 0.763 to 0.865). The corresponding AUC, along with sensitivity and specificity levels at the "best" thresholds for each machine-learning method, are thoroughly detailed in Table 2. The model selection process identified GBM as the initial frontrunner, deserving subsequent refinement and extensive testing.

**Table 2.** Area under the receiver operating characteristic curves for each machine-learning classifier run on the training set.

Model	Specificity	Sensitivity	AUC value
GBM	0.751 (0.684-0.818)	0.715 (0.650-0.781)	0.814 (0.763-0.865)
GLM	0.736 (0.673-0.798)	0.720 (0.642-0.798)	0.812 (0.769-0.854)
KNN	0.663 (0.592-0.731)	0.589 (0.505-0.672)	0.671 (0.622-0.719)
NB	0.429 (0.342-0.516)	0.871 (0.817-0.924)	0.812 (0.780-0.864)
NNET	0.611 (0.465-0.756)	0.701 (0.575-0.827)	0.688 (0.624-0.752)
RF	0.744 (0.689-0.830)	0.710 (0.630-0.777)	0.809 (0.766-0.851)
SVM	0.783 (0.732-0.833)	0.688 (0.621-0.765)	0.810 (0.763-0.856)

**Figure 3.**
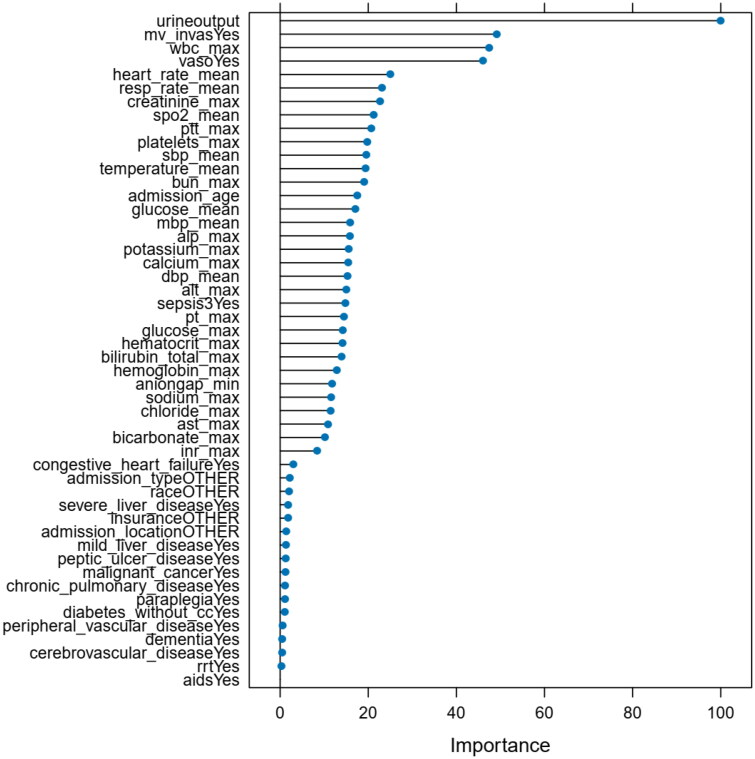
Variable importance of features included in gradient boosting machine algorithm for prediction of AKI. Variable importance is computed based on how important any given feature is to aid in the classification process when the classifier is built, determined by its effect on the performance measure. The greater the importance, the more essential the variable is to the performance of the model. Assumptions about effect size cannot be drawn directly about the relationship of variable importance to the primary outcome.

In the refinement of method-specific parameters, tuning was applied to the gradient boosting machine method, involving adjustments to the number of trees (ranging from 50 to 200), interaction depth (ranging from 1 to 8), shrinkage (ranging from 0.1 to 0.3), and the minimum number of variables at terminal nodes (ranging from 5 to 20). This process concluded with a finalized gradient boosting machine algorithm characterized by 50 trees, an interaction depth of 2, shrinkage of 0.1, and 10 minimum variables at terminal nodes. The final variable importance can be observed in Figure 3. The resulting model demonstrated an AUC of 0.816 (95% CI, 0.766 to 0.867).

**Figure 4.**
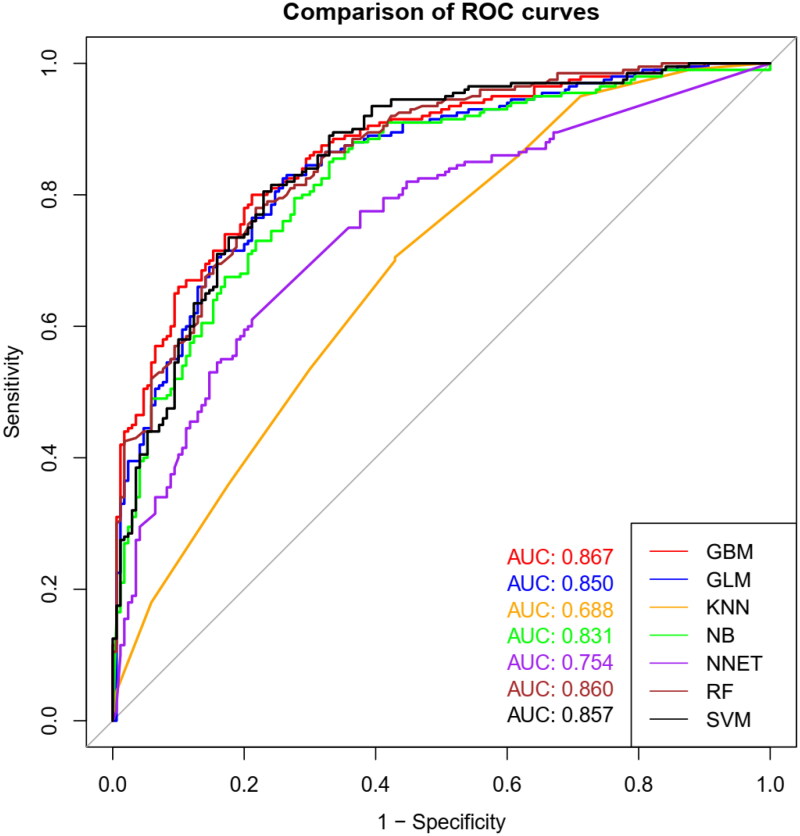
Receiver operating characteristic curves of machine-learning methods for prediction of AKI in the test data set. A greater area under the receiver operating characteristic curve represents higher discriminative ability of the model. Area under the receiver operative characteristics curves, as well as specificity and sensitivity of each machine learning model for prediction of AKI at “best” threshold are presented with 95% CIs. “best” threshold refers to the threshold at which specificity and sensitivity are both maximized.

The subsequent evaluation of the model on the test set yielded an AUC of 0.867 (95% CI, 0.831 to 0.903), a negative predictive value of 77% (95% CI, 76 to 78%), a positive predictive value of 82% (95% CI, 81 to 83%), and an overall classification accuracy of 79% (95% CI, 78 to 80%). The AUC values for all machine-learning classifiers executed on the test set are detailed in both Table 3 and Figure 4. The calibration plot of GBM model in test datasets illustrate in Appendix 2.

**Table 3.** Area under the receiver operating characteristic curves for each machine-learning classifier run on the test set.

Model	Specificity	Sensitivity	AUC value
GBM	0.788 (0.729-0.847)	0.800 (0.745-0.850)	0.867 (0.831-0.903)
GLM	0.741 (0.676-0.800)	0.825 (0.775-0.875)	0.849 (0.810-0.888)
KNN	0.571 (0.494-0.647)	0.705 (0.640-0.770)	0.688 (0.634-0.741)
NB	0.671 (0.600-0.735)	0.850 (0.800-0.895)	0.831 (0.790-0.872)
NNET	0.624 (0.553-0.694)	0.775 (0.715-0.835)	0.753 (0.704-0.802)
RF	0.782 (0.718-0.847)	0.780 (0.725-0.835)	0.859 (0.823-0.886)
SVM	0.771 (0.706-0.829)	0.805 (0.750-0.860)	0.856 (0.818-0.894)

## Discussion

The data from the MIMIC database was meticulously collected and utilized alongside a diverse array of machine-learning algorithms. This collaborative effort aimed to predict the likelihood of acute renal injury occurring within a seven-day window following admission to the ICU for patients diagnosed with acute pancreatitis. Our investigation resulted in the development of predictive models represented by area under the receiver operating characteristic curves, where the model employing the GBM technique emerged as the most prominent. Notably, the GBM model demonstrated the most robust performance, supported by AUC of 0.814 (95% CI, 0.763 to 0.865) for the training set and 0.867 (95% CI, 0.831 to 0.903) for the test set, respectively. This performance aligns well with our initial expectations.

Among the classical regression methodologies, generalized linear model (e.g. logistic regression) stands out as a pivotal tool for examining associations between AKI and relevant risk factors. For instance, Dongliang Yang et al. leveraged logistic regression to construct a predictive model with discerning efficacy in forecasting AKI and severe AKI in patients with mild and severe acute pancreatitis (MSAP and SAP). This model highlighted the significant importance of clinical parameters such as C-reactive protein, intra-abdominal pressure, and serum cystatin C in the prediction of AKI [19]. Similarly, Simin Wu et al. using multivariate logistic regression and a subsequent nomogram, demonstrated proficient predictive capability for early AKI occurrence in acute pancreatitis patients. The resulting nomogram achieved AUC of 0.795 (95% CI, 0.758–0.832) in the training cohort and 0.772 (95% CI, 0.711–0.832) in the validation cohort [7]. However, existing literature, as seen in certain studies [20, 21], suggests that conventional logistic regression may exhibit relatively modest performance indicators, quantified by AUC for receiver operating characteristic curves. Some studies also emphasize an elevated prediction error and comparative performance diminution compared to innovative techniques.

In recent times, the exploration of various machine learning algorithms, a subset of artificial intelligence involving the construction of predictive algorithms by ‘learning’ from data, has received increased attention. This methodology, inherently skilled at automated analysis of intricate datasets to yield substantive insights, has notably outperformed conventional statistical methods in terms of performance. This superiority arises from its ability to effectively decipher complex data patterns and generate meaningful outcomes. Notable contributions in this domain include the work of Yi Yang et al. who developed machine learning-based prediction models tailored for acute AKI, emphasizing the potential of random forest classifiers to enhance predictive efficacy in patients with acute pancreatitis [22]. Similarly, Yang Fei et al. demonstrated the utility of artificial neural networks (ANNs) in prognosticating the clinical risk for acute lung injury following severe acute pancreatitis (SAP) [23]. However, it is crucial to acknowledge the relatively modest sample sizes in these studies, limiting the achieved area under the curve values. A prominent contender in the landscape of machine learning algorithms is the gradient boosting machine, recognized for its precision and performance in predictive competitions. Its demonstrated attributes underscore its increasing prominence as a compelling alternative to conventional regression analyses, especially for predicting clinical adversities. In line with these trends, our findings affirm the superiority of the GBM model over alternative machine learning frameworks and traditional logistic regression models. The notable improvement in performance and heightened accuracy in predicting AKI among acute pancreatitis patients highlight the elevated potential of the GBM-based algorithm. This reinforces the prominence of gbm within the array of machine learning methodologies, affirming its status as a robust contender for enhancing predictive modeling outcomes in the context of clinical adverse events.

In line with these trends, our findings confirm the superiority of the GBM model over alternative machine learning frameworks and traditional logistic regression models. The notable improvement in performance and increased accuracy in predicting AKI among acute pancreatitis patients underscores the elevated potential of the GBM-based algorithm. This solidifies the prominence of GBM within the array of machine learning methodologies, reaffirming its status as a robust contender for enhancing predictive modeling outcomes in the context of clinical adverse events.

Through meticulous scrutiny of attribute significance within our model, we identified the pronounced influence of specific characteristics in predicting acute renal injury within the cohort of acute pancreatitis patients. Foremost among these determinants was urine volume, emerging as a pivotal factor, followed sequentially by invasive mechanical ventilation, white blood cell count, utilization of vasoactive drugs, mean heart rate, mean respiratory rate, and maximum creatinine levels. This aligns judiciously with the collective wisdom of diverse medical conditions, wherein variations in urine volume often foreshadow the emergence of acute renal injury [24]. It is essential to underscore that acute renal injury denotes a precipitous decrement in renal function, attributable to multifarious triggers, including ischemia, nephrotoxic agents, and infections [25]. Notably, a decline in urine volume signifies compromised renal perfusion and diminished glomerular filtration rate, portending the onset of acute renal injury.

Remarkably, our analysis unveiled the predictive significance of invasive mechanical ventilation in the context of AKI within acute pancreatitis. This observation aligns with earlier investigations [26]. It is discerned that acute respiratory failure stemming from acute pancreatitis necessitates recourse to invasive mechanical ventilation in ICU-admitted patients. This intervention, albeit essential, is recognized to potentially precipitate acute lung injury, exacerbating hypoxia and culminating in vasoconstriction, diminished renal perfusion, and reduced glomerular filtration rate. Notably, invasive mechanical ventilation instigates an elevation in intrathoracic pressure, inducing a concomitant reduction in venous return and mean arterial pressure, thereby fostering a milieu conducive to prerenal hypoperfusion and the subsequent onset of acute renal injury [27].

Cytokines, including IL-1β, IL-8, and IL-6, play a pivotal role in the potential pathogenesis of AKI. These mediators influence endothelial cells, leading to renal ischemia, thrombosis, and the release of oxygen free radicals [26]. Simultaneously, inflammatory mediators contribute to increased mucosal permeability and facilitate endotoxin translocation. Notably, endotoxin’s role in elevating endothelin levels orchestrates vasoconstriction, resulting in reduced renal blood flow and subsequent tubular necrosis, perpetuating the trajectory toward AKI development [27]. Importantly, this inflammatory milieu can intrinsically impede normative renal function, leading to a decrease in glomerular filtration rate and amplifying the risk of AKI [28]. A retrospective study [29] supports the utility of various biomarkers—hematocrit, platelets, leukocytes, lymphocytes, albumin, CRP, CRP/albumin ratio, neutrophil/lymphocyte ratio, procalcitonin, urea, and creatinine—evaluated at the point of hospital admission as effective prognostic indicators for AKI occurrence in acute pancreatitis patients. This observation aligns with the scholarly consensus, emphasizing the pivotal role of white blood cells in engendering inflammatory responses and supporting their significant contribution to AKI surveillance. It is noteworthy that the systemic inflammatory response, inherently interconnected with the AKI process, may result from localized inflammation within renal tissue [30].

The present study highlights the noticeable predictive capacity of vasoactive drugs in the context of acute pancreatitis-related acute kidney injury. In alignment with this observation, prior research has established that the need for mechanical ventilation, along with the use of vasopressor agents and renal replacement therapy, constitutes a cluster of risk factors associated with elevated mortality rates among AP patients [31]. It is crucial to emphasize that critically ill individuals require increased doses of vasopressor agents to manage blood pressure. Disturbances in heart rate and respiratory rate serve as indicators of changes in circulatory and respiratory domains, which, in turn, significantly impact renal functionality. It is important to recognize that deviations in circulatory and respiratory function lead to a cascade of events, consistently highlighting the impact on renal function [32].

However, this study is not without certain limitations. Firstly, it is essential to acknowledge its retrospective and single-center study. To enhance clinical applicability and achieve external validation, prospective efforts conducted across diverse centers are imperative. Furthermore, the model’s construction did not consider other significant factors, including the etiologies of acute pancreatitis, the stratification of acute pancreatitis severity, and variables related to intra-abdominal hypertension and abdominal compartment syndrome—factors that may influence the trajectory of AKI development in acute pancreatitis. Another limitation pertains to the relatively modest sample size underlying this inquiry, coupled with reliance solely on internal validation to assess the model’s precision and efficacy. To strengthen the generalizability and robustness of our findings, future investigations should embrace larger sample sizes and a more comprehensive incorporation of variables to validate our observations.

## Data Availability

The co-first author of this study, Wenbin Lu, has been granted authorization for data extraction from the MIMIC-IV database, specifically for research purposes (certification number: 50992435). The datasets used in the present study were downloaded from the following website: https://physionet.org/content/mimiciv/2.2/.
